# Gene expression data classification using topology and machine learning models

**DOI:** 10.1186/s12859-022-04704-z

**Published:** 2022-05-20

**Authors:** Tamal K. Dey, Sayan Mandal, Soham Mukherjee

**Affiliations:** 1 Department of Computer Science, Purdue University, West Lafayette, IN USA; 2 Department of Computer Science and Engineering, The Ohio State University, Columbus, OH USA

**Keywords:** Topological data analysis, Gene expression, Persistent cycles, Neural network

## Abstract

**Background:**

Interpretation of high-throughput gene expression data continues to require mathematical tools in data analysis that recognizes the shape of the data in high dimensions. Topological data analysis (TDA) has recently been successful in extracting robust features in several applications dealing with high dimensional constructs. In this work, we utilize some recent developments in TDA to curate gene expression data. Our work differs from the predecessors in two aspects: (1) Traditional TDA pipelines use topological signatures called barcodes to enhance feature vectors which are used for classification. In contrast, this work involves curating relevant features to obtain somewhat better representatives with the help of TDA. This representatives of the entire data facilitates better comprehension of the phenotype labels. (2) Most of the earlier works employ barcodes obtained using topological summaries as fingerprints for the data. Even though they are stable signatures, there exists no direct mapping between the data and said barcodes.

**Results:**

The topology relevant curated data that we obtain provides an improvement in shallow learning as well as deep learning based supervised classifications. We further show that the representative cycles we compute have an unsupervised inclination towards phenotype labels. This work thus shows that topological signatures are able to comprehend gene expression levels and classify cohorts accordingly.

**Conclusions:**

In this work, we engender representative persistent cycles to discern the gene expression data. These cycles allow us to directly procure genes entailed in similar processes.

## Background

The rapid advances in genome-scale sequencing have dispensed a comprehensive list of genes for different organisms. These data gives us a broad scope to comprehend the developmental and functional processes of these organisms. Since the advent of DNA microarray, it is now possible to measure the expression levels of large number of genes simultaneously. This has made a holistic analysis possible for gene data using their expression levels. The stochastic nature of biological processes and associated noise acquired during the mining process pose a fundamental challenge in modelling a mathematical structure explaining these high dimensional data. We look into two problems in data analysis involving gene expressions that are of current research interest.

A genome-wide association study (GWAS) is a method to link a subset of genes to a particular disease or physical phenomenon in an organism. It has been especially important to identify specific gene subsets not only from a clinical perspective but also from a data science perspective as well. The assimilation of these subsets enable better phenotype identification and improve prediction of cohort status using machine learning based approach. Our definition of cohort follows its common usage in biology where *a cohort is a group of animals of the same species, identified by a common characteristic, which are studied over a period of time as part of a scientific or medical investigation*. In our case all cohorts for each experiment belong to the same taxa.For small or medium sized data sets, since the number of gene expression in a cohort profile is far greater than the number of sample cohorts, disease prediction using neural networks is challenging as these architectures largely succeed when the number of samples is much larger. It becomes important for these cases to identify a subset of genes whose expression levels reflect the phenotype of the cohorts.

In addition, it is often the case that some cohort have incorrect or uncorrelated data due to instrumental or manual error. Hence, their gene expressions may not reflect their phenotype class. We find in practice that the elimination of such instances leads to better prediction scores and performance. In this work, we use topological data analysis to investigate both of these issues. We identify cohorts which are topologically relevant (Section Topo-Curated Cohort). We show that the use of these cohorts to determine phenotypes instead of the entire set improves classification. Next, in Topo-Relevant Gene Expression section, we look into the classic GWAS problem mentioned above to identify a small subset of genes by using topological data analysis. We compare classification results obtained by using this reduced gene subsets against the one obtained by using full gene pool. The results for the receded gene profile yields better prediction rate.

Topological data analysis (TDA), loosely speaking, explains the shape of a data using topological structures. Topological properties can be thought to remain invariant under continuous deformation. For instance, given a donut made of clay, topologically its shape remains the same if we stretch, twist, or bend it but changes if we cut or glue it. The theory of Algebraic Topology lays the mathematical foundation formalising this idea. Persistent Homology is a method to derive topological structures from a given data. Topological signatures, particularly based on Persistent Homology, enjoy some nice theoretical properties including robustness and scale invariance. These features are global and more resilient to local perturbations. This has made TDA an exciting area in data analysis with encouraging results in medical imaging [1, 2], protein analysis [3, 4], and molecular architecture [5, 6] among others. In previous works it has been shown that genes sharing similar attributes tend to cluster in high dimensions [7, 8]. This is because protein encoding genes that are part of the same biological pathway or have similar functionality are corregulated. This ultimately leads such gene clusters to have similar expression profiles. The property of clustering is essentially captured by the $zero^{th}$ order homology class in Persistent Homology (see next section). Motivated by these works, we are interested in finding if there exist relationships among similar genes in the higher order homology classes as well.

**Fig. 1:**
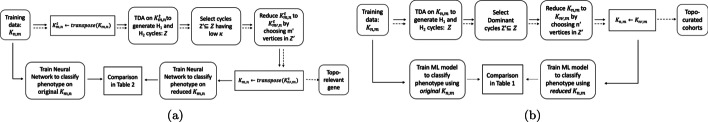
**a** Flowchart for *topo-relevant gene* expression extraction. Refer to Section Topo-Relevant Gene Expression for details. **b** Flowchart for *topo-curated cohort* extraction. Refer to Section Topo-Curated Cohort for details. In both, bold lines show the path to take for training or testing large data. Dotted lines used in Fig. 2

Traditional TDA pipelines use Persistent Homology to compute a set of intervals called barcodes which are used as topological feature in subsequent processing such as learning [3, 9–11]. While such barcodes provide robust topological signatures for the persistent features in data (such as tunnels, voids, loops, cycles etc.), their association to data is not immediately clear thus missing some crucial information. In effect, since these intervals represent homology classes, they contain the set of all loops around a topological hole. Thus using barcodes, it is hard to localize a feature, e.g., the shortest cycles or holes in a Persistent Homology class. This, in turn, hinders getting any direct mapping between the topological signatures and input cohorts or genes. So far there had been few studies addressing the problem of localizing persistent features and it has been shown that finding shortest cycles in given Persistent Homology classes is an NP-hard problem [12, 13]. However, taking advantage of the recent results in [12, 13], we are able to to compute good representative cycles for our application. These cycles capture definitive geometric features and provide a mapping between two domains of gene expression and topology.

**Fig. 2:**
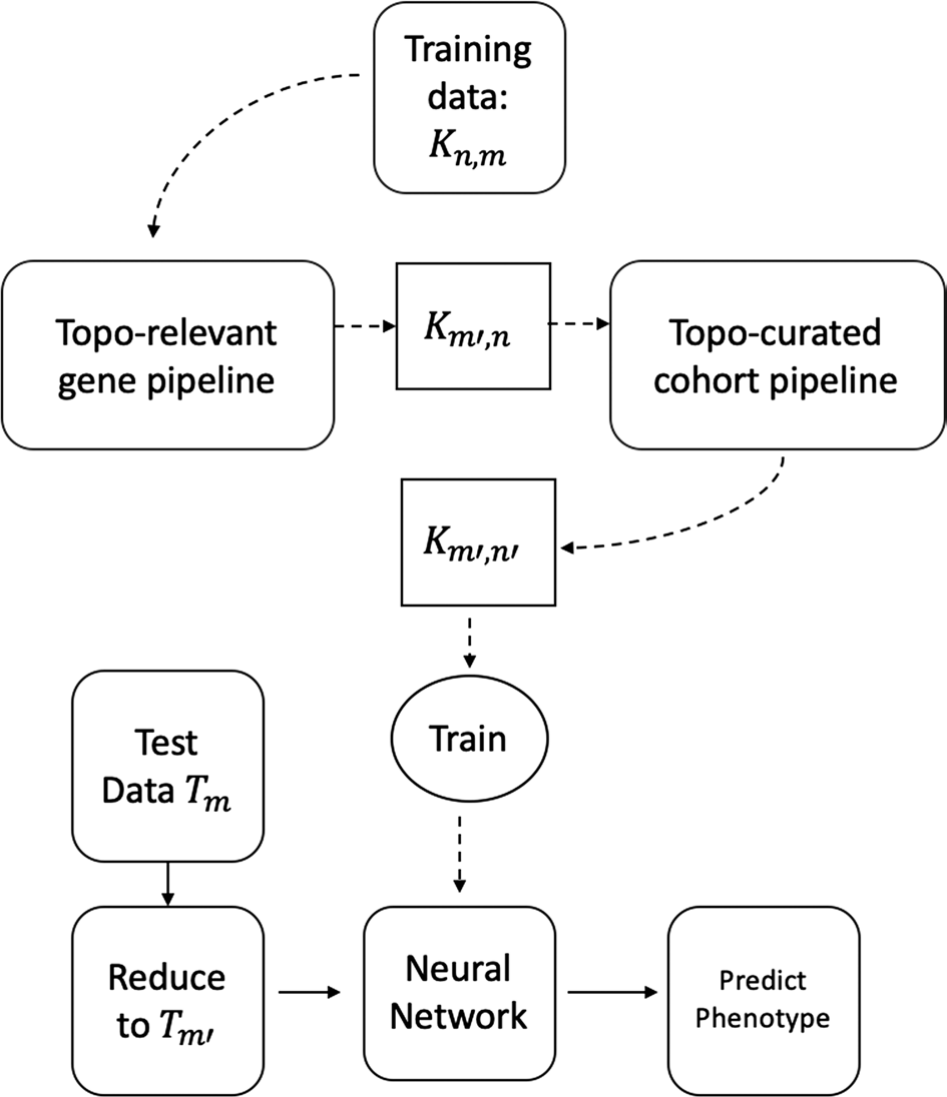
Flowchart of proposed pipeline. For units *Topo-relevant gene pipeline* and *Topo-curated cohort pipeline* we follow the dotted lines in Fig. 1a and b respectively

**Fig. 3:**
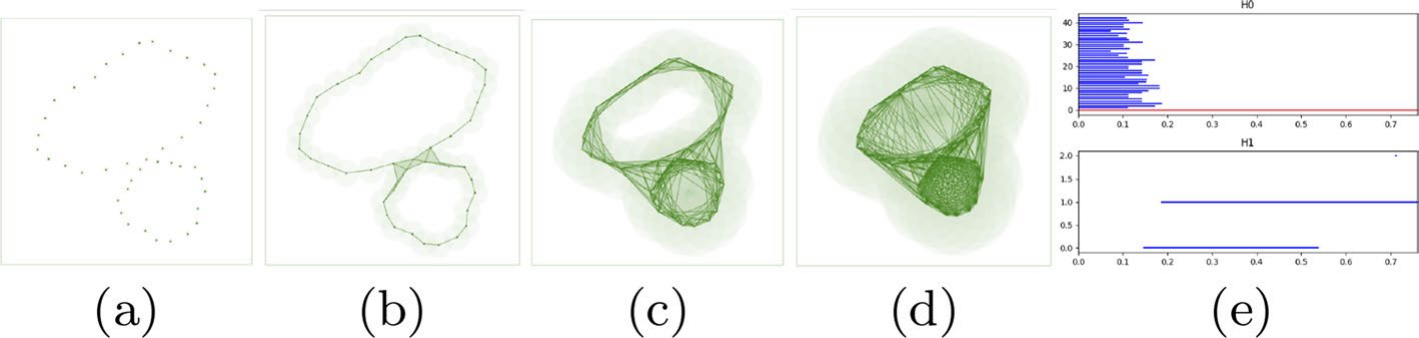
Illustration of persistent homology. Here **e** represents the barcodes (in fig **e** Y-axis is No. of cycles and X-axis is radius r) associated with increasing radius from **a** to **d**. [Taken from [10] with authors’ permission.]

In this paper we conduct two main experiments using the representative cycles: one to extract topologically relevant genes and the other to curate relevant cohorts. For these studies, some organisms were control units while others were either infected and/or injected with some antigen. The input consists of a matrix ${\mathcal {K}}$ which has *n* rows signifying the cohorts and their corresponding gene expressions in *m* columns. For obtaining and classifying topologically relevant (topo-relevant) genes, our experiment follow the pipeline in Fig. 1a whereas for determining curated cohorts, it follows the pipeline in Fig. 1b. For a large data set, we can trim out both insignificant cohorts and genes starting from the ‘Training data ${\mathcal {K}}_{n,m}$’. This can be done following the pipeline in Fig. 2. We train our neural network architecture on the final curated dataset and thereby test against any unknown cohort. For our experiments, we work on gene expressions extracted from different organisms including Drosophila, *Mus muculus*, and *Homo sapiens*. We convert these data into a binary or multi-classification problem based on their phenotype and feed it into the pipeline. Our methodology and results have been listed in Computing Topological Signature of Gene-Expression Data section (Fig. 3).

### Related works

In [14], gene expressions from peripheral blood data was used to build a model based on TDA network model and discrete Morse theory to look into routes of disease progression. These results on viruses suggest that Persistent Homology can be also used to study different forms of reticulate evolution. Topological structures have been used to analyse viruses by Emmet et al. [15]. They worked on influenza to show a bimodality of distribution of intervals in the persistent diagram. This bimodality indicates two scales of topological structure, corresponding to intra-subtype (involving one HA subtype) and inter-subtype (involving multiple HA subtypes) viral reassortments. Persistent Homology has also been used to identify DNA copy number aberrations [16]. Their experiments found a new amplification in 11q at the location of the progesterone receptor in the Luminal A subtype. Seemann et al. [17] used Persistent Homology to identify correlated patient samples in gene expression profiles. Their work focuses on the ${\mathcal {H}}_0$ homology class which is used to partition the point cloud. The paper by Nicolau et al. [18] identified a subgroup of breast cancers using topological data analysis in gene expressions.

Several works [19, 20] on use of machine learning techniques on gene expression profile have shown promising results. Kong et al. [21] used random forests to extract features for their Neural Network architecture. They investigate a problem similar to our *‘Topo-relevant gene’* and the results show significant improvement. [22] analyzes gene expression data to classify cancer types. Different techniques of supervised learning are used to understand genes and classify cancer. The authors of [23] use machine learning to identify novel diagnostic and prognostic markers and therapeutic targets for soft tissue sarcomas. Their work shows overlap of three groups of tumors in their molecular profile.

### Our contribution

We provide a technique based on persistent cycles (introduced in [12, 13]) to curate cohort data (datapoints) and gene expressions (features) (See Topo-Curated Cohort and Topo-Relevant Gene Expression sections). Through the experiments we show that these geometric structures, i.e. *cycles*, encode important information about the cohorts as choosing these *topo-curated cohorts* improve a classifier’s accuracy. In a separate experiment, we demonstrate that choosing these *topo-curated gene expressions* provide a better classification. In a way, we provide empirical evidence that there is a one-to-one correspondence between topological features and important gene functionality.

## Results

We now discuss the two ways to reduce the input ${\mathcal {K}}_{n,m}$ into ${\mathcal {K}}_{n'm'}$ where $n'\le n$ and $m'\le m$. The first section deals with finding pertinent cohorts, and the next with finding pertinent genes. In each subsection we describe the relevant procedure followed by results.

### Topo-curated cohort

For our first proof of concept, we find a subset of cohorts who provide topologically relevant information for classification. The aim is to remove cohorts having either incorrect or uncorrelated data due to instrumental or manual error. Specifically, given ${\mathcal {K}}_{n,m}$, we would like to find ${\mathcal {K}}'_{n',m}\subseteq {\mathcal {K}}_{n,m}$ for $n\le n'$ which improves classification odds for the cohorts. This subset of $n'$ cohorts should therefore be topologically more relevant. We start by converting the matrix ${\mathcal {K}}_{n,m}$ into a point cloud. This point cloud has *n* points each of dimension *m*. Hence each cohort in the matrix is converted to an *m*-dimensional point where each dimension represents the expression level for each gene. We use Sparse Rips on the resulting point cloud to obtain a simplicial complex ${\mathcal {K}}$ and its filtration (${\mathcal {F}}$) and apply the theory of Persistent Homology to obtain the set of finite intervals.

**Fig. 4:**
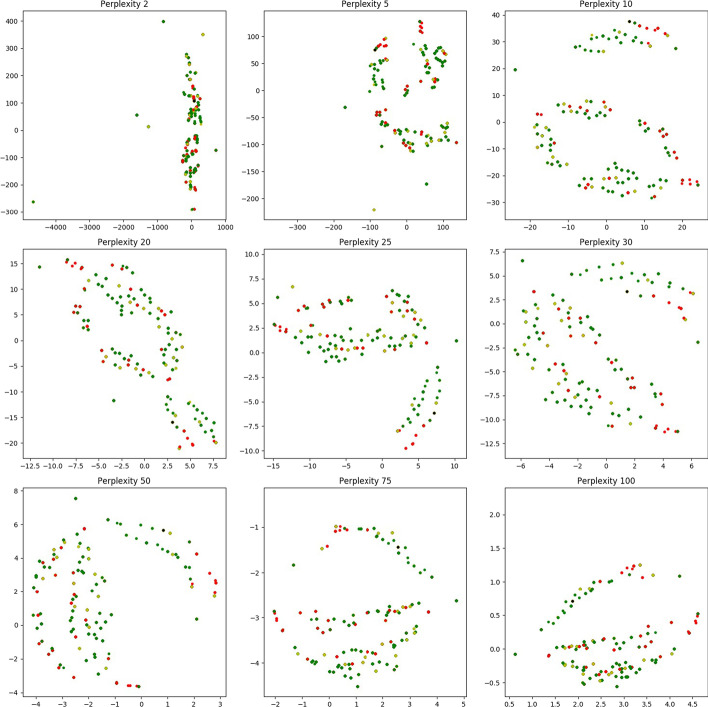
Plot of geodesic centers for dominating cycles using t-SNE. Red vert: non dominating cycles. Graded green points: dominating cycles. Alpha values indicate ratio of dominating phenotype in each cycle versus the other labels.Range of perplexity values in the t-SNE plot indicate the uniformity of topological cycles

We consider the dataset ${\mathbb {D}}0$ having three phenotypes. We generate the longest 100 ${\mathcal {H}}_2$ cycles based on their interval length ($\delta -\beta$). For each cycle, we consider the constituent vertices and their corresponding phenotype labels (${\mathcal {X}}$). We plot the count of ${\mathcal {X}}$ values in individual ${\mathcal {H}}_2$-cycles in Fig. 11a with the X, Y and Z axis representing ${\mathcal {X}} \in 0, 1,\text { and }2$ respectively. The black points indicate cycles where all vertices belong to a single phenotype. The red, green, and blue points indicate cycles having labels $(0,1),(0,2)\text { and }(1,2)$ respectively. The yellow points correspond to cycles having all three labels 0, 1,  and 2. The takeaway from this plot is that, since most points are skewed towards some particular axis, most ${\mathcal {H}}_2$-cycles have constituent vertices who belong predominantly to some particular label in ${\mathcal {X}}$. Thus topological cycles in general are inclined towards some ${\mathcal {X}}$ labels without any supervision as they were not fed with the phenotype labels. Note that we added a small random noise to each point coordinate to illustrate multiplicity. Figure 11b plots similar values for the top 200 ${\mathcal {H}}_2$ cycles for dataset ${\mathbb {D}}1$. Since this dataset has two phenotypes, we get a 2*d* plot. The red labels denote cycles which have an equal constituent phenotypes, whereas blue and cyan represent skew, with blacks representing single labeled cycles as before. As is evident, most cycles exhibit a predominance in either ${\mathcal {X}} \in \ 0\ or\ 1$. Based on the intuition of this plot, we define a cycle ${\mathcal {Z}}$ as a ***Dominant Cycle*** if, there exists a vertex set $U\subseteq {\mathrm Vert} ({\mathcal {Z}})$^1^ so that every vertex in *U* has the same label and $|U|\ge |{\mathrm Vert}({\mathcal {Z}})/2|$^2^.

**Fig. 5:**
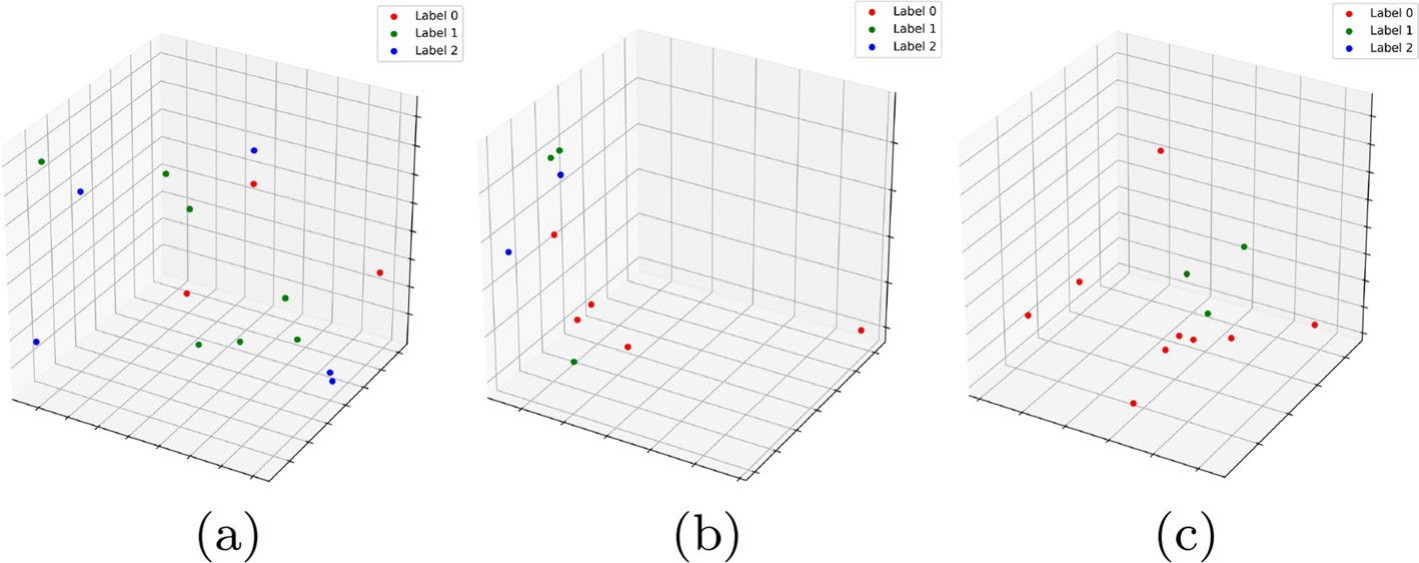
Three figures plotting individual dominating cycles for gene dataset ${\mathbb {D}}0$. These cycles actually reside in *m*-dimensions and are projected down to 3D using Principal component analysis. The colors indicate cohort phenotype labels ${\mathcal {X}}\in 0,1,2$. **a** Cycle 1 spanned by 15 vertices with 3 vertices having label 0, 7 vertices having label 1 and 5 with label 2. **b** Cycle 2 spanned by 10 vertices with 5 vertices having label 0, 3 vertices having label 1 and 2 with label 2. **c** Cycle 3 spanned by 12 vertices with 9 vertices having label 0, 3 vertices having label 1

To illustrate the frequency of dominating cycles versus non-dominating ones, we plot the geodesic centers of the ${\mathcal {H}}_2$ cycles for ${\mathbb {D}}0$ by projecting them down to 2d using T-sNE (Fig. 4). Red vertices indicate non dominating cycles while each of the graded green points indicate the dominating ones. Clearly, most of the topology cycles are dominating and indicate a vote towards some phenotype class. The alpha values (denoted by the green bar at the right) indicates the ratio of the dominating phenotype in each cycle versus the other labels (${\mathcal {X}}$). Hence, intuitively, more opaque a given point is, more is it dominated by a single class phenotype. Finally, we plot some of the individual dominating ${\mathcal {H}}_2$ cycles along with their phenotype labels in Fig. 5. Note that these points are part of the original D0 cohort point cloud and they were projected down to 3D using PCA.

#### Classification using machine learning

We work on several gene expression data extracted from different organisms. On each of these, we create a classification problem as described in the data section. For each dataset, we use the entire cohort list (irrespective of their phenotype) as an $(n\times m)$ dimensional point cloud. We generate the top 100 ${\mathcal {H}}_1$ and ${\mathcal {H}}_2$ cycles and select the *dominant cycles*. Next we select the vertices contained in these dominant cycles which form our new set of $n'(\le n)$-cohorts. Taking gene expression for these $n'$-cohorts lets us form our new smaller matrix ${\mathcal {K}}'_{n',m}$. Thereafter, we train supervised classification models once using ${\mathcal {K}}_{n,m}$ and then again using ${\mathcal {K}}'_{n',m}$ and compare results for each. We use 10-fold cross validation by splitting the data randomly into $80{-}20\%$ in each fold. For our classification models, we use two probability based classification models: Decision Tree and Naive Bayes.Note that we are interested in finding out whether the TDA pipeline can curate and retain the faithful representation of data. As a result we are comparing the performance of Decision Tree and Naive Bayes classifier on Topo-curated data. We do not report Support Vector Machine (SVM) result as its accuracy is too low to report. In general, probability based classification fared better than kernal based (SVM) techniques, hence we have reported our results on the same. The average value of accuracy, precision, and recall for the 10-fold cross validation is reported in Table 4. The column ‘FULL’ represents training on ${\mathcal {K}}_{n,m}$ while ${\mathcal {H}}_1+{\mathcal {H}}_2$ represent the union of $n'$ topo-relevant cohorts obtained from the dominant cycles in either ${\mathcal {H}}_1$ or ${\mathcal {H}}_2$. We also get good classification statistics for the vertices in dominant cycles picked up only by ${\mathcal {H}}_2$ cycles only as reported in the same table. As is evident from the results, reduction in the number of cohorts leads to an increase in classification measures. Thus TDA is able to pick up cohorts who carry more decisive gene expression levels for their individual phenotype classes.

### Topo-relevant gene expression

Our next problem is to reduce the matrix ${\mathcal {K}}_{n\times m}$ to ${\mathcal {K}}'$ of dimension $(n\times m')$ where $m'\ll m$. We use the persistent cycle descriptors ${\mathcal {H}}_1$ and ${\mathcal {H}}_2$ introduced in the previous section to extract $|m'|$ meaningful genes ($\mathcal {G'}$) such that $\mathcal {G'}\subset {\mathcal {G}}$. To this effect, we use the annotation of the gene set ${\mathcal {G}}$ based on their functional classification obtained from the *‘Panther Classification System by Geneontology’* [24] and the *‘NCBI Gene Data set’* [25]. Thus for each $g \in {\mathcal {G}}$, $\exists f: g\rightarrow R$, where *R* is a vector of functional attributes obtained from [24].

Once we obtain the representative cycles, we find the maximal cover of each cycle defined as follows:

*Maximal cover of representative cycle (*$\kappa$**)** For each gene expression $g\in {\mathrm{Vert}}({\mathcal {Z}})$ represented as vertices in a single representative cycle, we have a set of annotations *f*(*g*). We select the minimum set consisting of at least one annotation for each $g\in Vert({\mathcal {Z}})$. Let ${\mathcal {S}}$ be any set of annotations which contains at least one annotation for each $g\in \mathrm{Vert}({\mathcal {Z}})$. Thus,

$$\begin{aligned} \kappa = inf\{ |{\mathcal {S}}| \, | \,\forall g \in {{\mathrm{Vert}}}({\mathcal {Z}}), {\mathcal {S}} \cap f(g)\not =\emptyset \} \end{aligned}$$

**Fig. 6:**
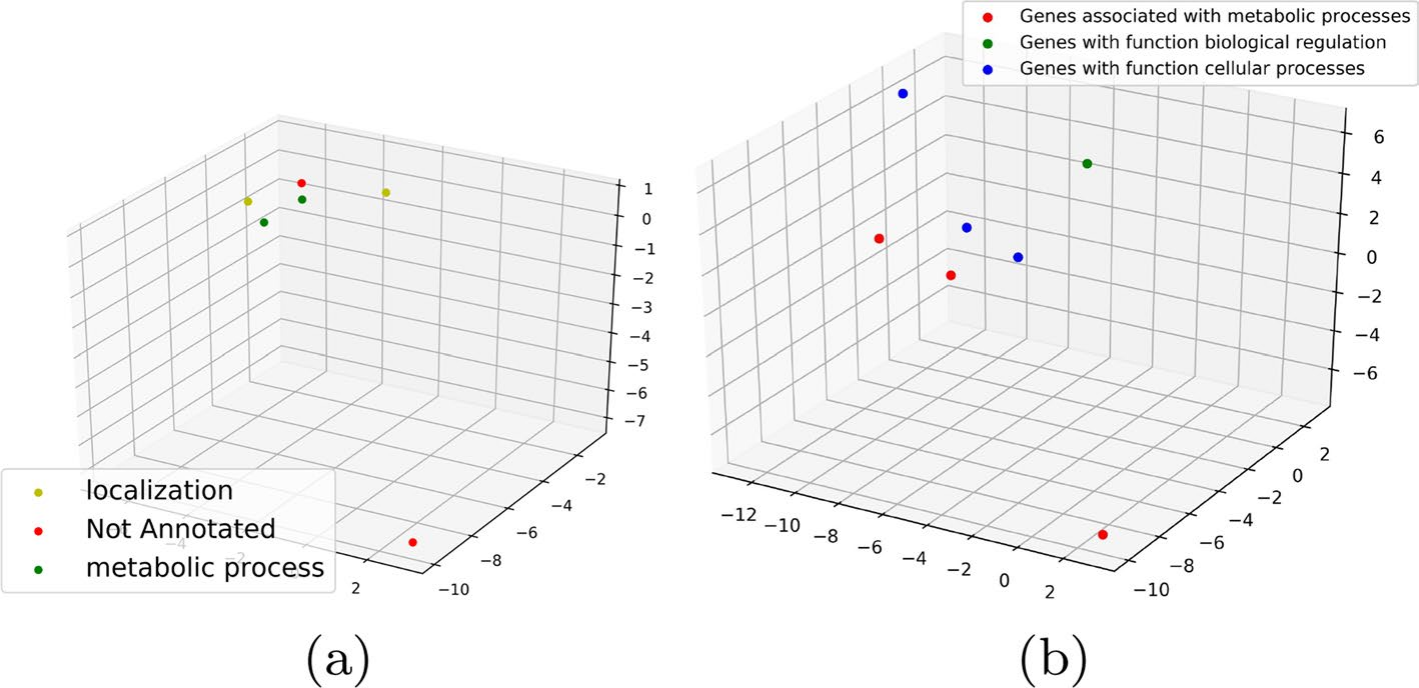
Three 2-cycles for gene dataset ${\mathbb {D}}6$ with colors indicating gene function labels. Total number of colors indicate the $\kappa$ value of the cycle. Note that a gene can be responsible for several functionalities. The legend in this plot takes into account only a single functionality which contributes towards the maximal cover of the representative cycle. **a** Low $\kappa$:3 **b** High $\kappa$:6

The idea behind using $\kappa$ is to get a sense of the functionality of the gene. A gene may be responsible for multiple processes described in the Panther and NCBI database. If $\kappa$ is low or unity for a certain ${\mathcal {Z}}$, it probably indicates that the gene expressions involved in ${\mathcal {Z}}$ reflect the functionality captured by $\kappa$. This is illustrated in Fig. 6 where we plot some of the ${\mathcal {H}}_2$ cycles generated on $\mathcal {K'}$ with color annotated by their functionality. We use PCA as before to project the points down to 3-dimensions. The three figures illustrate three instances of different $\kappa$-values. Consider the example in Fig. 6a for getting the intuition behind $\kappa$. The six vertices representing genes in the ${\mathcal {H}}_2$ cycles have function annotations: {1: Localization, 2: Not annotated, 3: Metabolic process, Cellular process, 4: Metabolic process, Cellular process, Biological regulation, 5: Metabolic process, Cellular process, Localization, 6: Not Annotated}. Out of this the set: {Localization, Not annotated, and Metabolic process} covers all the vertices and hence $\kappa$ is 3.

We choose ${\mathcal {C}}$ with low $\kappa$ values and select their component genes as part of $\mathcal {G'}$. We can control the size of $\mathcal {G'}$ based on the value of $\kappa$.

For all our experiments, we run each architecture and obtain performance measures on ${\mathcal {K}}$ which contains the exhaustive set of *m*-genes. We re-run these experiments on our trimmed set $\mathcal {K'}$ containing $m'(\ll m)$ topologically significant genes. Note that we may use the topo-relevant cohort extraction to additionally reduce $\mathcal {K'}_{n'\times m}$ into $\mathcal {K''}_{n'\times m'}.$ But since the public datasets we work on as our proof of concept have much less number of data to work with a Neural Net architecture, we do not trim the dataset. The results are in Table 1.

**Table 1:** Neural network result

Data Name	Human dengue (#:4415 )		Human bone marrow (#: 469)		Human bowel disease (#: 1745)	
Method	$TP({\mathcal {Z}})$	Full	$TP({\mathcal {Z}})$	Full	$TP({\mathcal {Z}})$	Full
# genes	1937	60619	5464	17258	1801	54715
Tr-Loss$(e^{-2})$	5.95	10.06	05.16	05.70	13.11	9.58
Tr-Acc	97.84	96.64	99.72	99.15	96.63	97.56
Tr-F1	97.86	96.48	99.72	99.15	96.60	97.55
Tr-Prec	97.86	96.48	99.72	99.15	96.60	97.55
Tr-Rec	97.86	96.48	99.72	99.15	96.60	97.55
Ts-Loss $(e^{-2})$	21.99	14.55	06.34	51.30	84.29	83.73
Ts-Acc	93.21	91.65	97.46	95.76	90.10	89.62
Ts-F1	92.26	90.67	96.95	95.74	90.34	89.66
Ts-Prec	93.48	90.67	96.95	95.74	90.34	89.66
Ts-Rec	93.48	90.67	96.95	95.74	90.34	89.66

The column $TP({\mathcal {Z}})$ indicates the results on reduced gene set using topology. Full indicates results on the full gene set. Tr-Loss, Tr-Acc, Tr-F1, Tr-Prec,Tr-Rec is loss, accuracy, F1-score, precision, and recall on the training data. Whereas the prefix Ts- indicate the same on the test set

### Neural network architecture

**Fig. 7:**
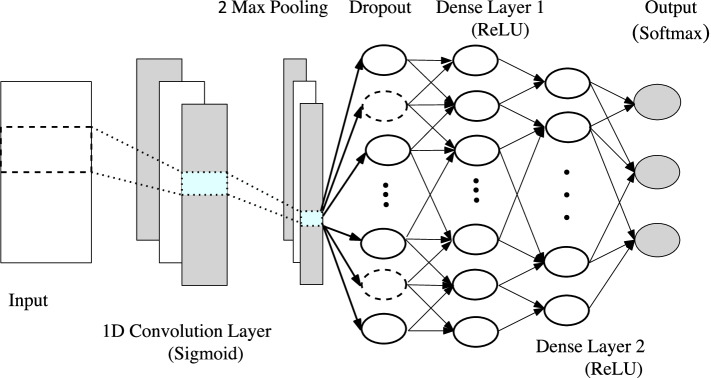
Neural network architecture. Number of filters, filter size, dropout probability, number of units in dense layers are hyperparameters and are tuned for different datasets

We use one dimensional convolutional neural network to perform experiments on gene-expression data. Our architecture is inspired by [21] who have managed to detect ‘relevant’ features of gene-expression data. The authors use a series of dense networks connected by activation functions. Since we provide some functional relevance among the genes, we sort them by their functionality and feed them to an additional convolutional layer on top of the model (Fig. 7). We start with this 1D-Convolutional Neural Network (CNN) layer activated by the sigmoid function. Sigmoid is a traditional activation function which provides a smooth non linearity in the network and since the architecture is not too deep, we do not need to worry about its shortcomings like the vanishing gradient. This is followed by a max pooling of size 2 and subsequently a dropout layer. This layer is connected to two densely connected layers with decreasing sizes. These layers have ReLU (Rectified Linear Unit) as their activation function as used in the paper by [21]. In the end, we add a softmax activation layer to determine the final label of the data. The hyper-parameters of the network can be tuned using advanced hyper-parameter optimization algorithm such as Bayesian Optimization. However, since this study is a proof of concept, and its purpose is to show the effectiveness of our feature selection, we fine tune them using manual observation.

**Fig. 8:**
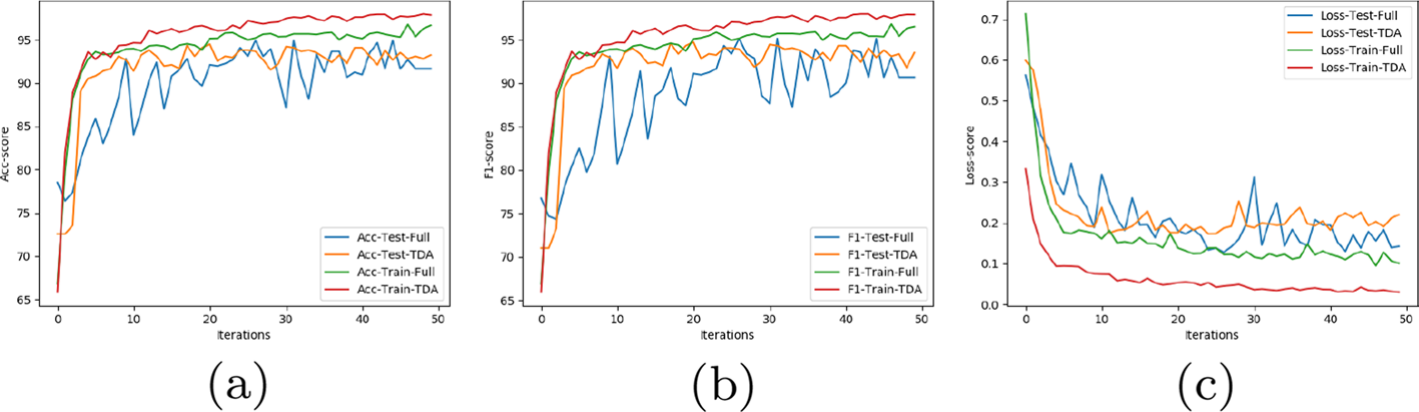
Comparison of **a** Accuracy, **b** F1-score, **c** Loss function for 50 epochs. For the TDA curated data, red and yellow lines represent train and test scores respectively. For the full data, they are represented by the green and blue lines

Since the number of samples is still less for CNN, overfitting is an issue. Notice that, for this precise reason, we do not curate this data using the pipeline in Topo-Curated Cohort section. Dropout layers are added after each layer to further prevent overfitting and reduce high variance. We, however, do not initiate early stopping as those pipelines are not amenable to orthogonalization. Finally, the model is optimised using Adam (Adaptive Moment Optimizer) [26]. The dataset is split evenly into 80-20 and cross validated for 50 epochs. The neural network has been implemented in Python using Tensorflow and Keras. The results for our experiment on datasets ${\mathbb {D}}5, {\mathbb {D}}6$ and ${\mathbb {D}}7$ is shown in Table 1. The row *# genes* show that our architecture using vertices selected from topological cycles are less than $30\%$ the size of the original gene pool. The results have, however, improved in all the cases. For experiments with Neural networks we follow the trend of the loss, accuracy and F1 score by plotting their value after every epoch in our algorithm. Figure 8 shows this result on dataset ${\mathbb {D}}7$. We see that the loss function on test data has been slightly higher but smoother than the full dataset. Despite this, using TDA the accuracy and F1 score has consistently performed better in every iteration for both the training and test data.

## Discussions

### Comparison with baseline

We compare our *Topo curated cohort* with the following standard outlier detection, unsupervised clustering methods.

- Local Outlier Factor
- Density-based spatial clustering of applications with noise [27] (DBSCAN)

It is noteworthy for the Droso breeding dataset DBSCAN fails to cluster and reports all the cohorts as outliers (Table 2). With DBSCAN as outlier detector and Decision Tree as a classifier, classifier’s accuracy reaches upto $100\%$ which is probably due to imbalance in the dataset and overfitting. In Table 2 we report the maximum accuracy obtained by our method, *i.e.* $max(Acc_{H1}, Acc_{H1+H2}, Acc_{H2})$ from Table 4. We then compare our *Topo-relevant gene expression* method with the following feature selection methods (See Table 3).

- Variance thresholding: Removes the low variance features that provide little information for modelling. [28–30].
- Select K-best features: Selects k-features according to the highest scores. The scoring function used is F-value from *analysis of variance* (ANOVA). [29, 30]
- Principal Component Analysis (PCA): Although this is a dimensionality reduction technique and not a feature selection method we incorporate it because PCA is used widely while analyzing high-dimensional data such as gene expression [31].
- Uniform Manifold Approximation and Projection for Dimension Reduction (UMAP): UMAP is a manifold learning learning and TDA based dimensionality reduction technique. UMAP assumes the data to be uniformly distributed on a Riemannian manifold and finds a low dimensional embedding by building a ‘fuzzy topological’ structure on it [32].

We use the same neural net architecture described in Neural Network Architecture section and report the test-set accuracy after selecting the features with the aforementioned feature selection methods and compare with our *Topo-Relevant Gene Expression* procedure. We report the test-accuracy only because we have observed during the training that the datasets are prone to overfitting (Table 4).

**Table 2:** Comparison with standard outlier detection and unsupervised clustering methods

Dataset	Decision tree			Naive Bayes		
	Local outlier factor	DBSCAN	Topo-curated cohort	Local outlier factor	DBSCAN	Topo-curated cohort
Droso breeding	32.14	–	**79.34**	**57.14**	–	42.24
Droso parasitod	36.84	100	**83.50**	47.36	**66.67**	–
Mouse E Coli	30.91	21.05	**89.29**	40.00	21.05	**59.21**
Mouse prion	35.89	39.53	**61.62**	39.74	39.53	**57.84**
Mouse liver cancer	46.67	56.21	**72.95**	60.00	56.52	**72.32**
Human bowel disease	17.74	19.41	**51.09**	48.39	**53.40**	–

Bold values indicate the highest accuracy obtained for different classifiers and datasets under consideration

**Table 3:** Comparison with standard feature selection methods

Dataset	Variance threshold	Select K-best	PCA	UMAP	Topo-relevant gene
Human dengue	92.26	93.41	74.21	85.39	**97**.**84**
Human bone marrow	95.37	92.25	76.50	**100**.**00**	99.72
Human bowel disease	61.62	61.69	61.62	61.63	**96**.**63**

The bold values indicate highest accuracy obtained for the feature selection methods

## Conclusions

We investigated into a topological technique to extract relevant cohorts and gene expressions so as to improve feature selections. Both our test cases show that the data follow topological alignment due to which the representative cycles covers the subset of vertices that are able to faithfully represent the data. As a result we are able to fit our training models better and reduce variance thereby getting better accuracy and f1-score. In future work, we will try to further tune our models so as to correlate the selected features with their functionality. For instance, there are cycles with low $\kappa$ values that have unannotated genes as its constituent vertices. It would be interesting to study functionalities of such specific genes with the other genes in a cycle.

## Methods

In this section we briefly describe the idea of Persistent Homology and their representative cycles. Since the idea is involved, the readers are directed to [33, 34] for more details on Persistent Homology. The representative cycles have been described in [12, 13]. Notice that, for curating cohorts, we convert the input $n\times m$ cohort-gene matrix to a point cloud of *n* points in *m* dimensions by treating each cohort as a point. Similarly, for curating gene expression we convert the transposed matrix to a point cloud of *m* points in *n* dimension with each gene as a single point. Our goal is to compute ‘good’ representative cycles in the Persistent Homology classes defined on a scaffold called ‘filtration’ built on top of these point clouds. Using these representative cycles, we identify the cohorts that are predominately present in a cycle and eliminate those which are not dominant in any of these cycles. Similar curation is done for gene expressions using the point cloud representing them.

### Persistence signature of point cloud data

We start with a point cloud data in any *n*-dimensional Euclidean space. These will essentially be *n*-dimensional points describing individual gene expressions (or cohorts). To illustrate the theory of Persistent Homology, we consider a toy example of taking a set of points in two dimensions sampled uniformly from a two-hole structure (Fig. 3). We start growing *n*-balls around each point, increasing their radius *r* continually and tracking the behavior of the union of these growing balls. Starting from *r=0* (Fig. 3a), we notice that at some $r=r_1$ (Fig. 3b) both holes are prominent in the union of ball structure. Increasing *r* further (Fig. 3d), we fill the smaller hole followed by the larger ones. During the change in the structure of the union of balls due to increase in radius, the larger of the two holes *‘persists’* for a larger range of *r* compared to the smaller one. Hence features that are more prominent are expected to persist for longer periods of increasing *r*. This exemplifies the basic intuition for topological persistence.

**Fig. 9:**
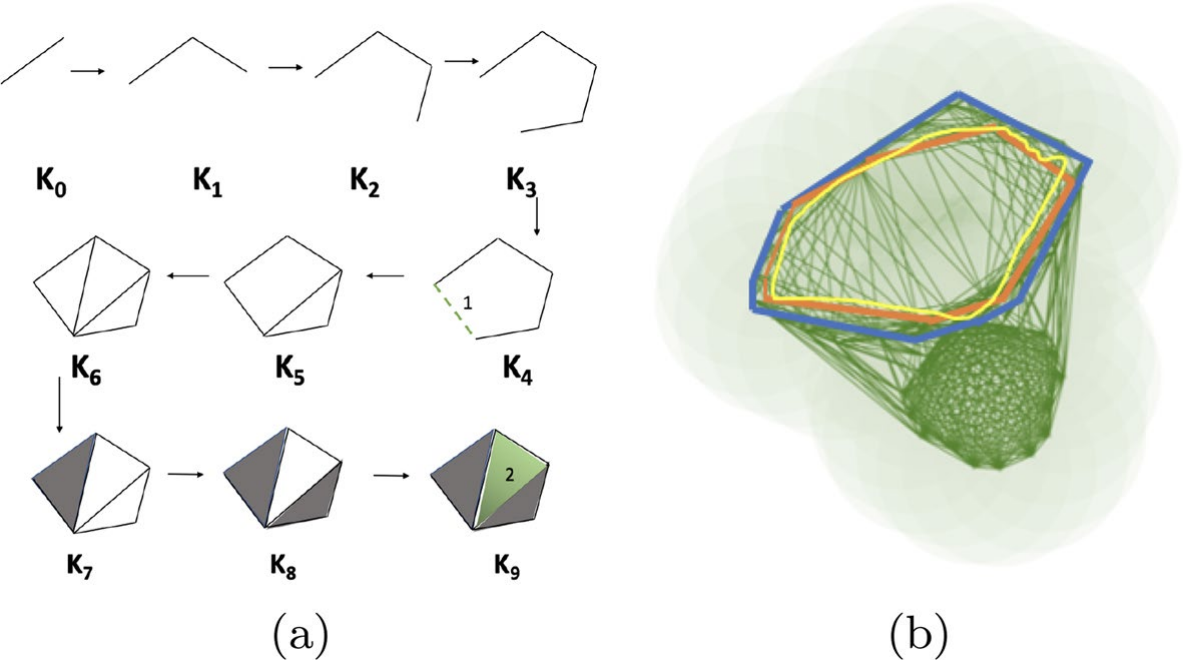
**a** Filtration ${\mathcal {F}}=K_0,..,K_9$ explaining persistence pairing. The edge inserted in ${\mathcal {K}}_4$ dies when the green triangle in ${\mathcal {K}}_9$ appears. **b** Different ${\mathcal {H}}_1$ cycles for same homology class

The persistence of holes are captured by a set of *birth-death* pairs (intervals) of homology classes that indicate at which value of *r* the class is born and where it dies. Each of these pairs is visualized using horizontal line segment known as a bar which together form the barcode [35] (Fig. 3e). The rank of a Persistent Homology group called the persistent Betti number captures the number of persistent features. This means, for the ${zero}^{th}$ homology group ${\mathcal {H}}_0$ consisting of ${zero}^{th}$ homology classes, $betti_0$ counts the number of connected components that arise in the filtration. For ${\mathcal {H}}_1$, $betti_1$ counts the number of *circular* holes (loops) being born as we proceed through the filtration. Similarly for ${\mathcal {H}}_2$, $betti_2$ gives a count of the number of surfaces enclosing three dimensional voids in the data. Thus, the short blue line segments of $betti_0$ in (Fig. 3e) correspond to the separate components that are joined to form one big connected component corresponding to the red line. The two long blue line segments of $betti_1$ correspond to the two holes in the structure, the largest representing the bigger hole.

For computational purposes, the growing sequence of the union of balls is converted to a growing sequence of triangulations, simplicial complexes in general, called a *filtration* (Fig. 9). The topological signatures are born when a series of say, edges (1-simplices), are connected to form a cycle and die when they are filled in with triangles (2-simplices). If we take the example in Fig. 9a, the theory of Persistent Homology suggests that in the filtration ${\mathcal {F}}=K_0\rightarrow K_1\rightarrow \ldots \rightarrow K_n=K$, the edges inserted in ${\mathcal {K}}_4$, ${\mathcal {K}}_5$ and ${\mathcal {K}}_6$ (1-simplex denoted $\sigma ^1_4$, $\sigma ^1_5$ and $\sigma ^1_6$ respectively)are the creators as introduction of which create class of homology cycles. We can think the creators as the representative of the cycles. By convention when a triangle appears in the filtration it kills the youngest homology class and is denoted by pairing with creators. In ${\mathcal {K}}_7$ the triangle kills the cycle created by the edge that came in ${\mathcal {K}}_6$. So, it pairs with $\sigma ^1_6$. In ${\mathcal {K}}_8$ the triangle pairs with $\sigma ^1_7$ and the big hole (created by $\sigma ^1_4$ and is the youngest creator unpaired) is filled up and destroyed by the last triangle (2-simplex denoted $\sigma ^2_9$) inserted in ${\mathcal {K}}_9$. Thus $\sigma ^1_4$ is paired with $\sigma ^2_9$ for interval [4, 9). The problem with relying only on the barcodes is that they tell us when the classes are born or die given a filtration. But for each homology class, there can be several cycles in the same class (Fig. 9b). Ideally, we would like the tightest cycle (blue one) in the class to be a representative cycle for a given bar. However, it is shown in [12] that computing such cycles even for ${\mathcal {H}}_1$ is an NP-Hard problem. A follow up paper [13] shows that for dimensions $\ge 1$, the problem remains NP-Hard. We therefore, use alternate polynomial time algorithms to build good representative ${\mathcal {H}}_1$ and ${\mathcal {H}}_2$ cycles given any barcode interval $[\beta ,\delta )$. The first algorithm [12] computes a good but not necessarily the tightest representative cycles. The second algorithm [13] computes the tightest representative cycles but for a specific class of domains called pseudo-manifolds. We briefly describe these two algorithms. 
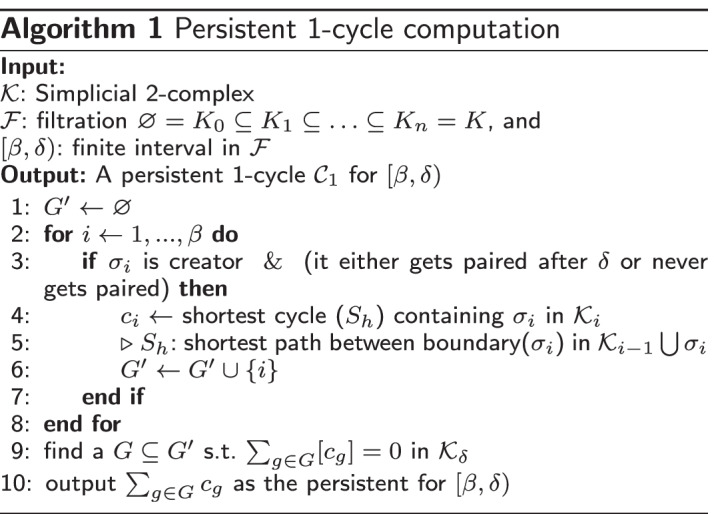


Algo. 1 generates ${\mathcal {H}}_1$ cycles. The idea is briefly as follows: we know that at the birth time $\beta$ of a 1-cycle (found by the persistence algorithm), an edge $\sigma ^1_\beta$ is inserted in ${\mathcal {F}}$ to form a cycle in ${\mathcal {K}}_\beta$. We hence check for the shortest path between the vertices of $\sigma ^1_\beta$ in ${\mathcal {K}}_{i-1}$ before $\sigma ^1_\beta$ is inserted. Since we know that at least one cycle *containing* $\sigma ^1_\beta$ is formed at ${\mathcal {K}}_\beta$, adding $\sigma ^1_\beta$ to this path gives us the shortest cycle at ${\mathcal {K}}_\beta$. At $\delta$, we need to know which cycle belonging to the homology class has died. This can be a linear combination of any cycles still alive including the cycle found at ${\mathcal {K}}_\beta$. This is found using a strategy of annotations [4]. In fact, it is shown in the paper that the shortest cycle found at ${\mathcal {K}}_\beta$ is exactly the shortest cycle for the interval in most practical cases.

Algo. 2 is used to compute ${\mathcal {H}}_2$ cycles for an interval $[\beta ,\delta )$ and can be extended to any ${\mathcal {H}}_n$. We first construct an undirected dual graph *G* for ${\mathcal {K}}$ where vertices of *G* are dual to 2-simplices of ${\mathcal {K}}$ and edges of *G* are dual to 1-simplices of ${\mathcal {K}}$. One dummy vertex termed as *infinite vertex* which does not correspond to any 2-simplices is added to *G* for graph edges dual to the boundary 1-simplices. We then build an undirected flow network on top of *G* where the source is the vertex dual to the death of an interval and the sink is the infinite vertex along with the set of vertices dual to those 2-simplices which are added to ${\mathcal {F}}$ after $\delta$. If a 1-simplex is $\sigma _\beta ^1$ or added to ${\mathcal {F}}$ before $\sigma _\beta ^1$, we let the capacity of its dual graph edge be its weight; otherwise, we let the capacity of its dual graph edge be $+\infty$. Finally, we compute a minimal cut of this flow network and return the 2-chain dual to the edges across the minimal cut as a minimal persistent cycle for the interval. The readers may consult the respective papers for ${\mathcal {H}}_1$-cycles [12] and ${\mathcal {H}}_2$-cycles [13] computations for more details.

Since computation of $H_n$-cycles is computationally expensive, especially in higher dimensions, we restrict ourselves with the computation of upto ${\mathcal {H}}_2$-cycles for our experiments. Most previous works on TDA had mainly included ${\mathcal {H}}_1$ intervals, with applications in gene expression being restricted to ${\mathcal {H}}_0$, so we hope to shed some new light into the problem even with this restricted setup. 
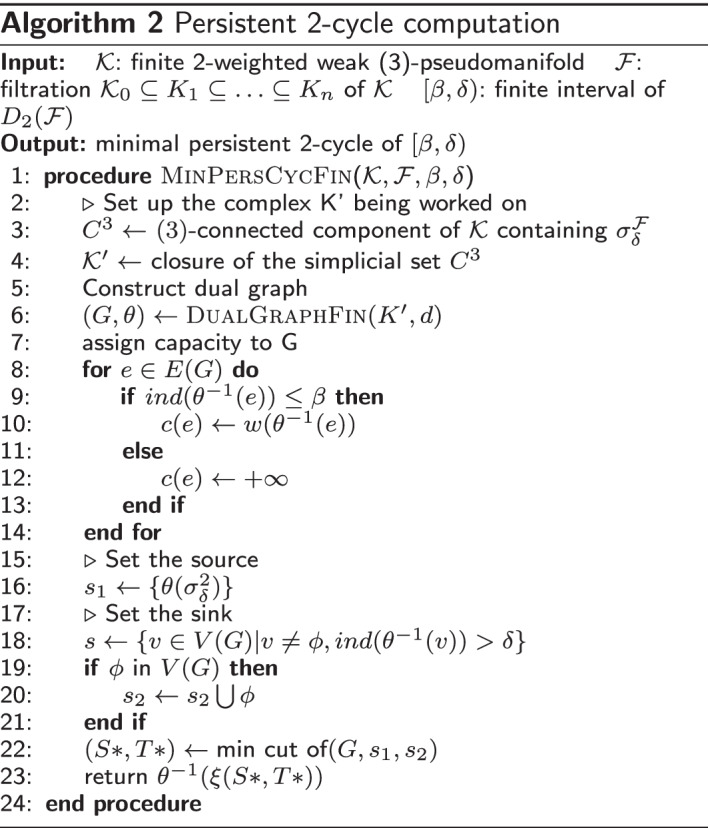


### Computing topological signature of gene-expression data

We work under the hypothesis that topological data analysis extracts relevant information sufficient for cohort classification. We note that topological feature extraction methods used in earlier works may not work in this setting. Traditionally, for many applications in bio science (say protein classification) and engineering, we find corresponding topological signatures using Persistent Homology for each sample (in this case cohorts or genes). These signatures are appended to the feature vectors. However, in this case, since each cohort is represented by a single 1D vector of gene expression levels, we are not able to find suitable signatures to append. This is why the algorithms we described in the previous section comes handy, as we will see in this section. We use our tools in two separate set of experiments. For algorithms 1 and  2, we need a simplicial complex ${\mathcal {K}}$, a filtration ${\mathcal {F}}$, and finite intervals. For all the studies in the paper, we use Sparse Rips [36] to obtain the simplicial complex ${\mathcal {K}}$ and its filtration (${\mathcal {F}}$). We can apply the theory of Persistent Homology to obtain the set of all finite intervals. In addition, algorithm 2 requires a pseudo-manifold (${\widetilde{K}}$) instead of a regular simplicial complex ${\mathcal {K}}$. For our case, this means that all triangles ($d=2$-simplices) has at most two tetrahedrons ($d+1=3$-simplices) attached to it. We convert ${\mathcal {K}}$ into ${\widetilde{K}}$ by allowing at most two cofaces (tetrahedra) per triangle which appear first in the filtration:

- Add all $\sigma ^{0\ldots d}$ to ${\widetilde{K}}$:
- $$\forall \sigma ^{d} \in K$$:
  - **Sort:** its co-faces ${\mathcal {T}} = \sigma ^{d+1}$ by ${\mathcal {F}}(\sigma ^{d+1})$
  - **If:** $|{\mathcal {T}}|\ge 2$, insert into ${\widetilde{K}}$, the first two $\sigma ^{d+1}$ in ${\mathcal {T}}$,
  - **Else:** insert ${\mathcal {T}}$ in ${\widetilde{K}}$

**Fig. 10:**
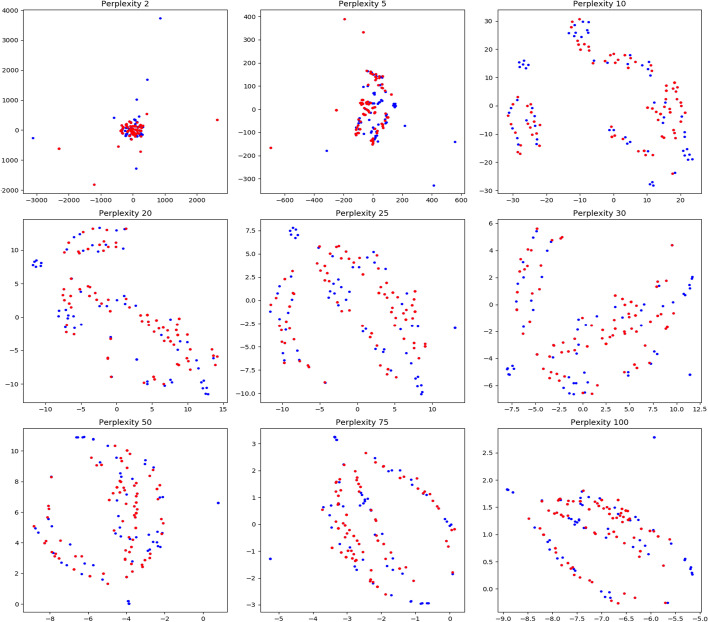
t-SNE on entire cohort point cloud (${\mathbb {D}}0$). Red vertices indicate cohorts included in top 100 ${\mathcal {H}}_2$ cycles whereas blue indicate otherwise. Note that different perplexity values for t-SNE indicate that the topological cycles are indeed uniform and not specific to a particular hyperparameter (perplexity) value

**Table 4:** Classification using topo-relevant cohort

EXPR	Decision tree				Naive Bayes classifier		
		FULL	H1+H2	H2	FULL	H1+H2	H2
Droso breeding	#	131	116	101	131	116	101
	Accuracy	0.714125	0.751768	0.793434	0.398146	0.412121	0.422444
	Precision	0.745417	0.815000	0.835000	0.389111	0.431756	0.451673
	Recall	0.712500	0.754167	0.795833	0.400000	0.416667	0.434478
Droso parasitod	#	89	85	51			
	Accuracy	0.792778	0.796667	0.811667	–	–	–
	Precision	0.817381	0.823571	0.859167	–	–	–
	Recall	0.792500	0.797500	0.825000	–	–	–
Mouse prion	#	321	292	168	321	292	146
	Accuracy	0.562310	0.616240	0.586843	0.555112	0.578489	0.576131
	Precision	0.562716	0.591471	0.543743	0.383462	0.378556	0.384572
	Recall	0.539712	0.564394	0.558267	0.415855	0.422354	0.423651
Mouse liver cancer	#	242	229	190	242	229	190
	Accuracy	0.682761	0.698934	0.729545	0.723232	0.723232	0.721404
	Precision	0.590716	0.579833	0.656051	0.444761	0.444761	0.412018
	Recall	0.573319	0.602582	0.641168	0.499837	0.499837	.506429
Mouse E.Coli	#	226	206	166	226	206	166
	Accuracy	0.880731	0.851794	0.892900	0.592770	0.592105	0.592105
	Precision	0.880541	0.853406	0.901481	0.604010	0.651101	0.652203
	Recall	0.868052	0.842963	0.891786	0.509841	0.511111	0.511111
Human bowel disease	#	1745	101	101			
	Accuracy	0.499698	0.510987	0.510987	–	–	–
	Precision	0.493808	0.509147	0.509147	–	–	–
	Recall	0.491258	0.501173	0.501173	–	–	–

Each of the data are explained in Dataset section. The # symbol indicates the size of each dataset. ‘–’ in the table means the stats were too low: the relevant classifier was unable to classify the given data. The column ‘FULL’ represents training on the full dataset while ${H}_1+{H}_2$ represent the union of $n'$ topo-relevant cohorts obtained from the dominant cycles in either ${H}_1$ or ${H}_2$ whereas $H_2$ represents cohorts obtained from the dominant cycles in $H_2$

### Dataset

We have a set of *n* cohorts (${\mathcal {C}}$) each represented by the gene expression profile of *m* genes (${\mathcal {G}}$). Thus our input is a matrix ${\mathcal {K}}$ of dimension $(n\times m)$ where each ${\mathcal {K}}_{i,j}$ represents the $j^{th}$ gene of the $i^{th}$ cohort. In addition, we have ${\mathcal {X}}: {\mathcal {C}}\rightarrow I$, where *I* is the phenotype for the cohort. For instance, ${\mathcal {X}}(c)=0$ may imply that c is healthy or control, whereas ${\mathcal {X}}(c)=1$ may imply they are infected or treated with an antigen depending on the experiment. Throughout our experiments we will work on several datasets containing gene expression profile of different organisms [37]. We provide a brief description of these data (Table 5).

($${\mathbb {D}}$$**0**):
*Droso Breeding* In this data set, the *Drosophila melanogaster* larvae is bred on a *Aspergillus nidulans* infested breeding substrate. The phenotypes differ on the different breeding condition for the Drosophilas. We assign label 0 to control, label 1 to the Drosophilas bred on *Aspergillus nidulans* mutant laeA, and label 2 to both the Drosophilas bred on wild Aspergillus nidulans and sterigmatocystin. Note that in this experiment, mutating laeA from wild *Aspergillus nidulans* removes sterigmatocystin production. Hence, both the wild Aspergillus and the class with external sterigmatocystin should have similar gene expression profile. The experiments in the dataset website confirms this fact, as there is no change in any gene expression profile between these two classes. The number of cohorts in the database is 131. Link: https://www.ebi.ac.uk/gxa/experiments/E-MTAB-5344/Results.
($${\mathbb {D}}$$*1*):
*Droso Parasitod* The data contains the profile of Drosophila larvae after a parasitod attack. There are two labels on the phenotype, one for the control and the other for the cohorts under parasitod attack. Thus, we have a binary classification problem in this case. Total cohorts count is 89. Link to this dataset: https://www.ebi.ac.uk/gxa/experiments/E-MAXD-6/Results.
($${\mathbb {D}}$$*2*):
*Mouse Prion* This data has *Mus musculus* as the cohort. The experiment investigates into the effects of two different strains of the prion disease. The phenotypes are ‘RML infected’, ‘301V infected’, and the healthy control which are assigned labels $0{-}2$ respectively. Total cohort count is 321. Link:https://www.ebi.ac.uk/gxa/experiments/E-MTAB-76/Results
($${\mathbb {D}}$$*3*):
*Mouse Liver Cancer* This is again a binary classification problem of the *Mus muculus*. The two phenotypes are control type and liver cancer cohorts. We take healthy control as 0 and mice treated with carcenogenic compunds as 1. Total cohort count is 242. Link: https://www.ebi.ac.uk/arrayexpress/experiments/E-GEOD-18858/.
($${\mathbb {D}}$$*4*):
*Mouse EColi.* The three phenotypes in this dataset are the *Eschreichia coli*, Staphylococcus, and control. The total number of cohorts across all three phenotypes in 226. Link: https://www.ebi.ac.uk/gxa/experiments/E-ENAD-29/Results.
($${\mathbb {D}}$$*5*):
*Human Bowel Disease* A binary classification problem where the phenotype are from cohorts suffering Crohns Disease and placebo cases. This is a big dataset having gene expressions of 1745 human. Link: https://www.ebi.ac.uk/gxa/experiments/E-GEOD-100833/Results
($${\mathbb {D}}$$*6*):
*Human Bone Marrow* This data set contains gene expressions of patients having bone marrow failure and cytogeneic abnormalities along with healthy cohorts who serve as control. This dataset has 469 cohorts. Link: https://www.ebi.ac.uk/arrayexpress/experiments/E-GEOD-32719/.
($${\mathbb {D}}$$*7*):
*Human Dengue* This is yet another big dataset having two types of phenotypes where we have gene expression of Dengue patients versus cohort control. Cohort count for this dataset is 4415. Link: https://www.ncbi.nlm.nih.gov/geo/download/?acc=GSE116672.

**Table 5:** Cohort count in each phenotype. Details of the label assignment for each dataset can be found in its description

Dataset	Phenotype	Samples per class	Total Samples
Droso breeding	Control	32	131
	Aspergillus	34	
	Aspergillus + Sterigmatosystin	65	
Droso parasitod	Control	45	89
	Under parasitod attack	44	
Mouse Prion	RML infected	154	321
	301V infected	122	
	Control	45	
Mouse liver cancer	Control	63	242
	With liver cancer	179	
Mouse E.Coli	With E.Coli	64	226
	With staphylococcus	102	
	Control	60	
Human Bowel Disease	Crohn’s disease	101	1745
	Placebo	1113	
	Control	531	
Human bone marrow	Bone marrow failure	391	469
	Control	78	
Human dengue	Dengue fever	3311	4415
	Contrrol	1104	

**Fig. 11:**
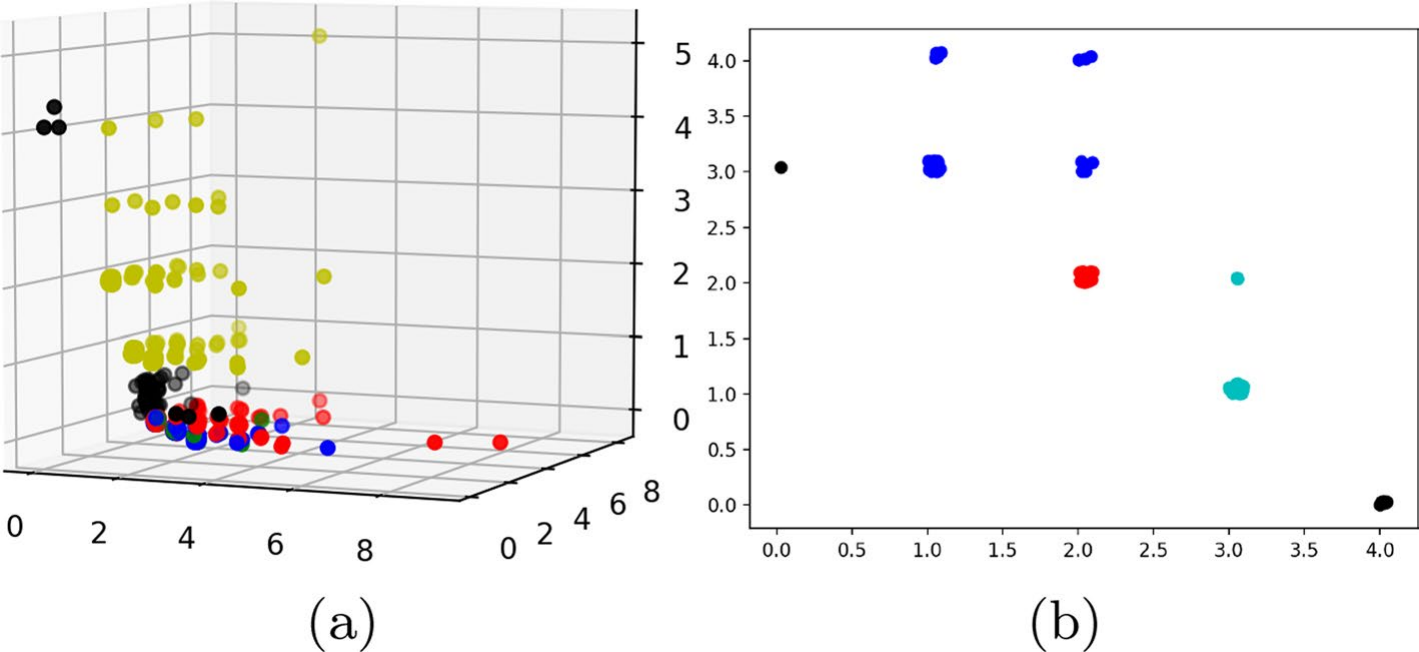
**a** Count of vertex labels in individual ${\mathcal {H}}_2$-cycles for ${\mathbb {D}}0$. The red points indicate cycles having phenotype labels 0 and 1, blue indicates cycles with labels 1 and 2 whereas green (very few in the top 500 ${\mathcal {H}}_2$ cycles) indicates labels 0 and 2. **b** Count of vertex labels in individual ${\mathcal {H}}_2$-cycles for ${\mathbb {D}}1$. Red indicate cycles having equal phenotype labelled vertices. Blue and cyan indicate prevalence of label 0 and 1 respectively. In both the diagrams, black points indicate cycles having a single phenotype label

Since each data point reside in dimension $> 3$ we apply t-Distributed Stochastic Neighbor Embedding (t-SNE) on ${\mathbb {D}}0$ Drosophila dataset to obtain a 2D projection for visualization in Fig. 10. To get a sense of the distribution of topological cycles, we calculate the top 100 representative ${\mathcal {H}}_2$ cycles based on their interval length ($\delta -\beta$). In Fig. 10, we color a cohort vertex red if it is contained in any of the top 100 ${\mathcal {H}}_2$ cycles. The cohorts not included are painted blue. This figure shows the uniform distribution of the topological cycles w.r.t the entire dataset (Fig. 11).

## Data Availability

The source code for computing persistent persistent 1-cycles can be found at https://github.com/Sayan-m90/Persloop-viewer whereas persistent 2-cycles is hosted in https://github.com/Sayan-m90/Minimum-Persistent-Cycles. The Neural Network Architecture and the Probabilistic ML (Machine Learning) approaches on top of it can be built with any standard open source ML libraries. Experiments done to compare with the standard approaches can be found https://github.com/soham0209/Gene-Expression. The datasets are available publicly. Link of the datasets are provided in their respective description.

## Abbreviations

TDA: Topological data analysis
ML: Machine learning
NCBI: National Center for Biotechnology Information
CNN: Convolutional neural network

## Footnotes

1 ${\mathrm Vert}({\mathcal {Z}})$ denotes the constituent vertices on the cycle ${\mathcal {Z}}$.

2 The modulo operator implies size of a set.
